# A Self-Organizing Incremental Spatiotemporal Associative Memory Networks Model for Problems with Hidden State

**DOI:** 10.1155/2016/7158507

**Published:** 2016-11-03

**Authors:** Zuo-wei Wang

**Affiliations:** Department of Computer Science and Software, Tianjin Polytechnic University, Tianjin 300387, China

## Abstract

Identifying the hidden state is important for solving problems with hidden state. We prove any deterministic partially observable Markov decision processes (POMDP) can be represented by a minimal, looping hidden state transition model and propose a heuristic state transition model constructing algorithm. A new spatiotemporal associative memory network (STAMN) is proposed to realize the minimal, looping hidden state transition model. STAMN utilizes the neuroactivity decay to realize the short-term memory, connection weights between different nodes to represent long-term memory, presynaptic potentials, and synchronized activation mechanism to complete identifying and recalling simultaneously. Finally, we give the empirical illustrations of the STAMN and compare the performance of the STAMN model with that of other methods.

## 1. Introduction

The real environment in which agents are is generally an unknown environment where there are partially observable hidden states, as the large partially observable Markov decision processes (POMDP) and hidden Markov model (HMM) literatures attest. The first problem for solving a POMDP is hidden states identifying. In many papers, the method of using the *k*-step short-term memory to identify hidden states has been proposed. The *k*-step memory is generally implemented through tree-based models, finite state automata, and recurrent neural networks.

The most classic algorithm of tree-based model is U-tree model [1]. This model is a variable length suffix tree model; however, this method can only obtain the task-related experiences rather than general knowledge of the environment. A feature reinforcement learning (FRL) framework [2, 3] is proposed, which considers maps from the past observation-reward-action history to an MDP state. Nguyen et al. [4] introduced a practical search context trees algorithm for realizing the MDP. Veness et al. [5] introduced a new Monte-Carlo tree search algorithm integrated with the context tree weighting algorithm to realize the general reinforcement learning. Because the depth of the suffix tree is restricted, these tree-based methods cannot efficiently handle long-term dependent tasks. Holmes and Isbell Jr. [6] first proposed the looping prediction suffix trees (LPST) in the deterministic POMDP environment, which can map the long-term dependent histories onto a finite LPST. Daswani et al. [7] extended the feature reinforcement learning framework to the space of looping suffix trees, which is efficient in representing long-term dependencies and perform well on stochastic environments. Daswani et al. [8] introduced a squared *Q*-learning algorithm for history-based reinforcement learning; this algorithm used a value-based cost function. Another similar work is by Timmer and Riedmiller [9], who presented the identify and exploit algorithm to realize the reinforcement learning with history lists, which is a model-free reinforcement learning algorithm for solving POMDP. Talvitie [10] proposed the temporally abstract decision trees to learning partially observable models. These *k*-step memory representations based on multidimensional tree required additional computation models, resulting in poor time performances and more storage space. And these models have poor tolerance to fault and noise because of the accurate matching of each item.

More related to our work, finite state automata (FSA) has been proved to approximate the optimal policy on belief states arbitrarily well. McCallum [1] and Mahmud [11] both introduced the incremental search algorithm for learn probabilistic deterministic finite automata, but these methods learn extremely slowly and with some other restrictions. Other scholars use recurrent neural networks (RNN) to acquire memory capability. A well known architecture for RNN is Long Short-term Memory (LSTM) proposed by Hochreiter and Schmidhuber [12]. Deep reinforcement learning (DRL) [13] first was proposed by Mnih et al., which used deep neural networks to capture and infer hidden states, but this method still apply to MDP. Recently, deep recurrent *Q*-learning was proposed [14], where a recurrent LSTM model is used to capture the long-term dependencies in the history. Similar methods were proposed to learn hidden states for solving POMDP [15, 16]. A hybrid recurrent reinforcement learning approach that combined the supervised learning with RL was introduced to solve customer relationship management [17]. These methods can capture and identify hidden states in an automatic way. Because these networks use common weights and fixed structure, it is difficult to achieve incremental learning. These networks were suited to resolve the spatiotemporal pattern recognition (STPR), which is extraction of spatiotemporal invariances from the input stream. For the temporal sequence learning and recalling in more accurate fashion, such as the trajectory planning, decision making, robot navigation, and singing, special neural network models for temporal sequence learning may be more suitable.

Biologically inspired associative memory networks (AMN) have shown some success for this temporal sequence learning and recalling. These networks are not limited to specific structure, realizing incremental sequence learning in an unsupervised fashion. Wang and Yuwono [18] established a model to recognize and learn complex sequence which is also capable of incremental learning but need to provide different identifiers for each sequence artificially. Sudo et al. [19] proposed the self-organizing incremental associative memory (SOIAM) to realize the incremental learning. Keysermann and Vargas [20] proposed a novel incremental associative learning architecture for multidimensional real-valued data. However, these methods cannot address temporal sequences. By using time-delayed Hebb learning mechanism, a self-organizing neural network for learning and recall of complex temporal sequences with repeated items and shared items is presented in [21, 22], which was successfully applied to robot trajectory planning. Tangruamsub et al. [23] presented a new self-organizing incremental associative memory for robot navigation, but this method only dealt with simple temporal sequences. Nguyen et al. [24] proposed a long-term memory architecture which is characterized by three features: hierarchical structure, anticipation, and one-shot learning. Shen et al. [25, 26] provided a general self-organizing incremental associative memory network. This model not only leaned binary and nonbinary information but realized one-to-one and many-to-many associations. Khouzam [27] presented a taxonomy about temporal sequences processing methods. Although these models realized heteroassociative memory for complex temporal sequences, the memory length *k* still is decided by designer which cannot vary in self-adaption fashion. Moreover, These models are unable to handle complex sequence with looped hidden state.

The rest of this paper is organized as follows: In Section 2, we introduce the problem setup, present the theoretical analysis for a minimal, looping hidden state transition model, and derive a heuristic constructing algorithm for this model. In Section 3, STAMN model is analyzed in detail, including its short-term memory (STM), long-term memory (LTM), and the heuristic constructing process. In Section 4, we present detailed simulations and analysis of the STAMN model and compare the performance of the STAMN model with that of other methods. Finally, a brief discussion and conclusion are given in Sections 5 and 6 separately.

## 2. Problem Setup

A deterministic POMDP environment can be represented by a tuple *E* = 〈*S*, *A*, *O*, *f*_*s*_, *f*_*o*_〉, where *S* is the finite set of hidden world states, *A* is the set of actions that can be taken by the agent, *O* is the set of possible observations, *f*_*s*_ is a deterministic transition function *f*_*s*_(*S*, *A*) → *S*, and *f*_*o*_ is a deterministic observation function *f*_*o*_(*S*, *A*) → *O*. In this thesis, we only consider the special observation function *f*_*o*_(*S*) → *O* that solely depends on the state *s*. A history sequence *h* is defined as a sequence of past observations and actions {*o*_1_, *a*_1_, *o*_2_, *a*_2_,…, *a*_*t*−1_, *o*_*t*_}, which can be generated by the deterministic transition function *f*_*s*_ and the deterministic observation function *f*_*o*_. The length |*h*| of a history sequence is defined as the number of observations in this history sequence.

The environment that we discuss is deterministic and the state space is finite. We also assume the environment is strongly connected. However the environment is deterministic, which can be highly complicated, and nondeterministic at the level of observation. The hidden state can be fully identified by a finite history sequence in a deterministic POMDP, which is proved in [2]. Several notations are defined as follows:

 trans⁡(*h*, *a*) = {*o*  ∣  *o* is a possible observation following *h* by taking action *a*}.
 
*γ*(*s*_*i*_, *d*_*ij*_) denotes the observations sequence *o*_1_, *o*_2_,…, *o*_*n*_ generated by taking actions sequence *d*_*ij*_ from state *s*_*i*_.

Our goal is to construct a minimal, looping hidden state transition model by use of the sufficient history sequences. First we present the theoretical analysis showing any deterministic POMDP environment can be represented by a minimal, looping hidden state transition model. Then we present a heuristic constructing algorithm for this model. Corresponding definitions and lemmas are proposed as follows.

**Definition 1 (identifying history sequence).**

A identifying history sequence *h*_*i*_ is considered to uniquely identify the hidden state *s*_*i*_. In the rest of this paper, the hidden state *s*_*i*_ is equally regarded as its identifying history sequence *h*_*i*_, so *s*_*i*_ and *h*_*i*_ can replace each other.

**Figure 1:**
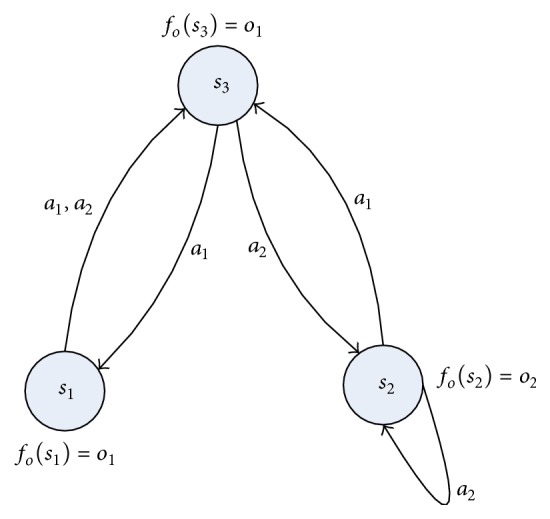
A simple example of deterministic POMDP.

A simple example of deterministic POMDP is illustrated in Figure 1. We conclude easily that an identifying history sequence *h*_2_ for *s*_2_ is expressed by {*o*_2_} and an identifying history sequence *h*_1_ for *s*_1_ is expressed by {*o*_2_, *a*_1_, *o*_1_, *a*_1_, *o*_1_}. Note that there may exist infinitely many identifying history sequences *h*_*i*_ for *s*_*i*_ because the environment is strongly connected and may exist as unbounded long identifying history sequences *h*_*i*_ for *s*_*i*_ because of uninformative looping. So this leads us to determine the minimal identifying history sequences length *k*.

**Definition 2 (minimal history sequences length *k*).**

The minimal identifying history sequences length *k* for the hidden state *s*_*i*_ is defined as follows: The minimal identifying history sequence *h* for the hidden state *s*_*i*_ is the identifying history sequence *h* such that no suffix *h*′ of *h* is also identified for *s*_*i*_. However, the minimal identifying history sequence may have unbounded length, because the identifying history sequence can include looping arbitrarily many times, which merely lengthens the history sequence. For example, two identifying history sequences *h*_1_ and *h*_1_′ for *s*_1_ are separately expressed to {*o*_2_, *a*_1_, *o*_1_, *a*_1_, *o*_1_} and {*o*_2_, *a*_1_, *o*_1_, *a*_1_, *o*_1_, *a*_1_, *o*_1_, *a*_1_, *o*_1_}. In this situation, {*o*_1_, *a*_1_, *o*_1_, *a*_1_, *o*_1_} is treated as the looped item. So the minimal identifying history sequences length *k* for the hidden state *s*_*i*_ is the minimal length value of all identifying history sequences by excising the looping portion.

**Definition 3 (a criterion for identifying history sequence).**

Given a sufficient history sequence, for the set of history sequences for the hidden state *s*, if each history sequence *h* for the hidden state *s* exists, which satisfies that trans(*h*, *a*) is the same observation for each action, thus we consider the history sequence *h* as identifying history sequence.  Definition 3 is correct iff a full sufficient history sequence is given.

**Lemma 4 (backward identifying history sequence).**

We assume an identifying history sequence *h* is for a single hidden state *s*, if *h*′ is a backward extension of *h* by adding an action *a* ∈ *A* and an observation *o* ∈ *O*; then *h*′ is a new identifying history sequence for the single state *s*′ = *f*_*s*_(*s*, *a*). *s*′ is called the prediction state of *s*.

**Proof**

We assume *h*′ is a history sequence {*o*_1_, *a*_1_,…, *a*_*t*−1_, *o*_*t*_} generated by the transition function *f*_*s*_ and observation function *f*_*o*_. And *h* is a history sequence {*o*_1_, *a*_1_,…, *a*_*t*−2_, *o*_*t*−1_} as a prefix of *h*′. Since *h* is identifying history sequence for *s*, it must be that *s*_*t*−1_ = *s*. Because the environment is deterministic POMDP, thus, the next state *s*′ is uniquely determined by *f*_*s*_(*s*_*t*−1_, *a*_*t*−1_) = *s*_*t*_ after the action *a*_*t*−1_ has been executed. Then *h*′ is a new identifying history sequence for the single state *s*_*t*_ = *f*_*s*_(*s*_*t*−1_, *a*_*t*−1_) = *f*_*s*_(*s*, *a*_*t*−1_) = *s*′.

**Lemma 5 (forward identifying history sequence).**

We assume an identifying history sequence *h*′ is for a single hidden state *s*′, if *h* is a prefix of *h*′ by removing the last action *a* ∈ *A* and the last observation *o* ∈ *O*; then *h* is the identifying history sequence for the single state s such that *s*′ = *f*_*s*_(*s*, *a*). s is called the previous state of *s*′.

**Proof**

We assume *h*′ is a history sequence {*o*_1_, *a*_1_,…, *a*_*t*−1_, *o*_*t*_} generated by the transition function *f*_*s*_ and observation function *f*_*o*_. And *h* is a history sequence {*o*_1_, *a*_1_,…, *a*_*t*−2_, *o*_*t*−1_} as a prefix of* h* by removing the action *a*_*t*−1_ and the observation *o*_*t*_. Since *h*′ is an identifying history sequence for the state *s*′, it must be that *s*_*t*_ = *s*′. Because the environment is deterministic POMDP, thus, the current state *s*′ is uniquely determined by the previous state *s* such that *f*_*s*_(*s*_*t*−1_, *a*_*t*−1_) = *s*′. Then *h* is the identifying history sequence for the state *s* such that *f*_*s*_(*s*_*t*−1_, *a*_*t*−1_) = *f*_*s*_(*s*, *a*_*t*−1_) = *s*′.

**Theorem 6 .**

Given the sufficient history sequences, a finite, strongly connected, deterministic POMDP environment can be represented soundly by a minimal, looping hidden state transition model.

**Proof**

We assume *E* = 〈*S*, *A*, *O*, *f*_*s*_, *f*_*o*_〉, where *S* is the finite set of hidden world states and |*S*| is the number of the hidden states. First, for all hidden states, at least a finite length identifying history sequence *h* for one state *s* exists; because the environment is strongly connected, there must exist a transition history *h*′ from *s* to *s*′, according to Lemma 4, by backward extending of *h* by adding *h*′, which is the identifying history sequence for *s*′. Since the hidden state transition model can identify the looping hidden state, there exists the maximal transition history sequence length from *s* to *s*′ which is |*S*|. Thus, this model has minimal hidden state space with |*S*|.

Since the hidden state transition model can correctly realize the hidden state transition *s*′ = *f*_*s*_(*s*, *a*), this model can correctly express all identifying history sequences for all hidden state *s* and possesses the perfect transition prediction capacity, and this model has minimal hidden state space with |*S*|. This model is a *k*-step variable history length model, and the memory depth *k* is a variable value for the different hidden state. This model is realized by the spatiotemporal associative memory networks in Section 3.

If we want to construct correct hidden state transition model, first we necessary to collect sufficient history sequences to perform statistic test to determine the minimal identifying history sequence length *k*. However, in practice, it can be difficult to obtain a sufficient history. So we propose a heuristic state transition model constructing algorithm. Without the sufficient history sequence, the algorithm may produce a premature state transition model, but this model at least is correct for the past experience. We realize the heuristic constructing algorithm by way of two definitions and two lemmas as follows.

**Definition 7 (current history sequence).**

A current history sequence is represented by the *k*-step history sequence {*o*_*t*−*k*+1_, *a*_*t*−*k*+1_, *o*_*t*−*k*+2_,…, *a*_*t*−1_, *o*_*t*_} generated by the transition function *f*_*s*_ and the observation function *f*_*o*_. At *t* = 0, the current history sequence *h*_0_ is empty. *o*_*t*_ is the observation vectors at current time *t*, and *o*_*t*−*k*+1_ is the observation vectors that precede *k*-step at time *t*.

**Definition 8 (transition instance).**

The history sequence associated with time *t* is captured as a transition instance. The transition instance is represented by the tuple *h*_*t*+1_ = (*h*_*t*_, *a*_*t*_, *o*_*t*+1_), where *h*_*t*_ and *h*_*t*+1_ are current history sequences occurring at times *t* and *t* + 1 on episode. A set of transition instances will be denoted by the symbol F, which possibly contains transitions from different episodes.

**Lemma 9 .**

For any two hidden states *s*_*i*_ and *s*_*j*_, *s*_*i*_ ≠ *s*_*j*_ iff trans(*s*_*i*_, *a*)≠trans(*s*_*j*_, *a*).

This lemma gives us a sufficient condition to determine *s*_*i*_ ≠ *s*_*j*_. However, this lemma is not a necessary condition.

*Proof by Contradiction.* We assume that *s*_*i*_ = *s*_*j*_; thus for all actions sequences *d*_*ij*_ = *a*_1_, *a*_2_,…, *a*_*n*_, there exists *γ*(*s*_*i*_, *d*_*ij*_) = *γ*(*s*_*j*_, *d*_*ij*_). *γ*(*s*_*i*_, *d*_*ij*_) = *γ*(*s*_*j*_, *d*_*ij*_) is contradicting with trans(*s*_*i*_, *a*)≠ trans(*s*_*j*_, *a*). Thus, original proposition is true.

**Lemma 10 .**

For any two identifying history sequences *h*_*i*_ and *h*_*j*_ separately for *s*_*i*_ and *s*_*j*_, there exists *h*_*i*_ ≠ *h*_*j*_ iff *s*_*i*_ ≠ *s*_*j*_. However, Lemma 10 is not a necessary condition.

*Proof by Contradiction.* Since an identifying history sequence *h* is considered to uniquely identify the hidden state *s*, the identifying history sequences *h*_*i*_ uniquely identify the hidden state *s*_*i*_ and the identifying history sequences *h*_*j*_ uniquely identify the hidden state *s*_*j*_. We assume *h*_*i*_ = *h*_*j*_; thus there must exit *s*_*i*_ = *s*_*j*_, which is contradicting with *s*_*i*_ ≠ *s*_*j*_. Thus, original proposition is true.

However, the necessary condition for Lemma 10 is not always true, because there maybe exist several different identifying history sequences for the same hidden state.

**Algorithm 11 (a heuristic constructing algorithm for the state transition model).**

The initial state transition model is constructed making use of the minimal identifying history sequence length *k* = 1. And the model is empty initially.

The transition instance *h* is empty, and *t* = 0:

(1) We assume the given model can perfectly identify the hidden state. The agent makes a step in the environment (according to history sequence definition, the first item in *h* is the observation vector). It records the current transition instance on the end of the chain of transition instance. And at each time *t*, for the current history sequence *h*_*t*_, execute Algorithm 12, if the current state *s* is looped to the hidden state *s*_*i*_; then go to step (2); otherwise a new node *s*_*i*_ is created in the model; go to step (4).
(2) According to Algorithm 13, if trans⁡(*h*_*t*_, *a*_*j*_) ≠ trans⁡(*h*_*i*_, *a*_*j*_), then the current state *s* is distinguished from the identifying sate *s*_*i*_; then go to step (3). If there exists trans⁡(*h*_*t*_, *a*_*j*_) = trans⁡(*h*_*i*_, *a*_*j*_), then go to step (4).
(3) According to Lemma 10, for any two identifying history sequences *h*_*i*_ and *h*_*j*_ separately for *s*_*i*_ and *s*_*j*_, there exists *h*_*i*_ ≠ *h*_*j*_ iff *s*_*i*_ ≠ *s*_*j*_. So the identifying history sequence length for *h*_*i*_ and *h*_*j*_ separately for *s*_*i*_ and *s*_*j*_ must increase to *k* + 1 until *h*_*i*_ and *h*_*j*_ are discriminated. We must reconstruct the model based on the new minimal identifying history sequence length *k*, and go to step (1).
(4) If the action node and corresponding *Q* function for the current identifying state node exist in the model, the agent chooses its next action node *a*_*j*_ based on the exhaustive exploration *Q* function. If the action node and corresponding *Q* function do not exist, the agent chooses a random action *a*_*j*_ instead, and a new action node is created in the model. After the action control signals to delivery, the agent obtains the new observation vectors by trans(*s*_*i*_, *a*_*j*_); go to step (1).
(5) Steps (1)–(4) continue until all identifying history sequences *h* for the same hidden state *s* can correctly predict the trans(*h*, *a*) for each action.

Note, we apply the *k*-step variable history length from *k* = 1 to *n*, and the history length *k* is a variable value for the different hidden state. We adopt the minimalist hypothesis model in constructing the state transition model process. This constructing algorithm is a heuristic. If we adopt the exhaustiveness assumption model, the probability of missing looped hidden state increases exponentially, and many valid loops can be rejected, yielding larger redundant state and poor generalization.

**Algorithm 12 (the current history sequence identifying algorithm).**

In order to construct the minimal looping hidden state transition model, we need to identify the looped state by identifying hidden state. Current history sequence with *k*-step is needed. According to Definition 7, current *k*-step history sequence at time *t* is expressed by {*o*_*t*−*k*+1_, *a*_*t*−*k*+1_, *o*_*t*−*k*+2_,…, *a*_*t*−1_, *o*_*t*_}. There exist three identifying processes.

(*1) k-Step History Sequence Identifying.* If the identifying history sequence for *s*_*i*_ is {*o*_*i*−*k*+1_, *a*_*i*−*k*+1_, *o*_*i*−*k*+2_,…, *a*_*i*−1_, *o*_*i*_}, satisfy

(1) $$\begin{array}{c} o_{t}=o_{i},\\ a_{t-1}=a_{i-1},\\ \vdots \\ o_{t-k+2}=o_{i-k+2},\\ a_{t-k+1}=a_{i-k+1},\\ o_{t-k+1}=o_{i-k+1}. \end{array}$$

Thus, the current state *s* is looped to the hidden state *s*_*i*_. In the STAMN, the *k*-step history sequence identifying activation value *m*_*j*_^*s*^(*t*) is computed.

(*2) Following Identifying.* If the current state *s* is identified as the hidden state *s*_*i*_, the next transition history *h*_*t*+1_ is represented by *h*_*t*+1_ = (*h*_*t*_, *a*_*t*_, *o*_*t*+1_) through trans(*s*_*i*_, *a*_*t*_). According to Lemma 4, the next transition history *h*_*t*+1_ is identified as the transition prediction state *s*_*j*_ = *f*_*s*_(*s*_*i*_, *a*_*t*_).

In the STAMN, the following activation value *q*_*j*_^*s*^(*t*) is computed. 

(*3) Previous Identifying.* If there exists a transition instance *h*_*t*+1_ = (*h*_*t*_, *a*_*t*_, *o*_*t*+1_), *h*_*t*+1_ is an identifying history sequence for state *s*_*j*_. According to Lemma 5, the previous state *s*_*i*_ is uniquely identified by *h*_*t*_ such that *s*_*j*_ = *f*_*s*_(*s*_*i*_, *a*_*t*_). So if there exists a transition prediction state *s*_*j*_, and *s*_*j*_ = *f*_*s*_(*s*_*i*_, *a*_*t*_), then the previous state *s*_*i*_ is uniquely identified.

In the STAMN, the previous activation value *d*_*j*_^*s*^(*t*) is computed.

**Algorithm 13 (transition prediction criterion).**

If the model correctly represents the environment model as Morkov chains, then, for all state *s*, for all identifying history sequence *h* for the same state, it is satisfied that trans(*h*, *a*) is the same observation for the same action.

If *h*_1_ and *h*_2_ are the identifying history sequences with *k*-step memory for the hidden state *s*, there exist trans(*h*_1_, *a*)≠trans(*h*_2_, *a*), according to Lemma 9; thus *s*_*i*_ is discriminative with *s*_*j*_.

In the STAMN, the transition prediction criterion is realized by the transition prediction value P_j_^s^(t).

## 3. Spatiotemporal Associative Memory Networks

In this section, we want to relate the spatiotemporal sequence problem to the idea of identifying the hidden state by a sequence of past observations and actions. An associative memory network (AMN) is expected to have several characteristics: (1) An AMN must memorize incrementally, which has the ability to learn new knowledge without forgetting the learned knowledge. (2) An AMN must be able to not only record the temporal orders but also record sequence items duration time with continuous time. (3) An AMN must be able to realize the heteroassociation recall. (4) An AMN must be able to process the real-valued feature vectors in bottom-up method, not the symbolic items. (5) An AMN must be robust and must be able to recall the sequence correctly with incomplete or noisy input. (6) An AMN can realize learning and recalling simultaneously. (7) An AMN can realize interaction between STM and LTM; dual-trace theory suggested that the persistent neural activities of STM can lead to LTM.

The first thing to be defined is the temporal dimension (discrete or continuous). The previous researches are mostly based on a regular intervals of Δ*t*, and few AMN have been proposed to deal with not only the sequential characteristic but also sequence items duration time with continuous time. However, this characteristic is important for many problems. In speech production, writing, music generation and motor-planning, and so on, the sequence item duration time and sequence item repeated emergence have essential different meaning. For example, “*B* ^4^” and “*B*-*B*-*B*-*B*” are exactly different, the former represents that item *B* sustains for 4 timesteps, and the latter represents that item *B* repeatedly emerges for 4 times. STAMN can explicitly distinguish these two temporal characteristics. So a history of past observations and actions can be expressed by a special spatiotemporal sequence *h*. *h* = {*o*_*t*−*k*+1_, *a*_*t*−*k*+1_, *o*_*t*−*k*+2_,…, *a*_*t*−1_, *o*_*t*_}, where *k* is the length of sequence of *h*; the items of *h* include the observation items and the action items, where *o*_*t*_ denotes the real-valued observation vectors generated by taking action *a*_*t*−1_. *a*_*t*_ denotes the action taken by the agent at time *t*, where *t* does not represent temporal discrete dimension by sampling at regular intervals Δ*t* but represents the *t*th step in the continuous-time dimension, which is the duration time between the current item and the next one.

A spatiotemporal sequence can classified as simple sequence and complex sequence. Simple sequence is a sequence without repeated items; for example, the sequence “*B*-*A*-*E*-*L*” is a simple sequence, whereas those containing repeated items are defined as the complex sequence. In complex sequence, the repeated item can be classified as looped items and discriminative items by identifying the hidden state, for example, in the history sequence “*X*-*Y*-*Z*-*Y*-*Z*”, “*Y*,” and “*Z*” maybe the looped items or discriminative items. Identifying the hidden state needs to introduce the contextual information resolving by the *k*-step memory. The memory depth *k* is not fixed and *k* is a variable value in different parts of state space.

### 3.1. Spatiotemporal Associative Memory Networks Architecture

We build a new spatiotemporal associative memory network (STAMN). This model makes use of neuron activity decay of nodes to achieve short-term memory, connection weights between different nodes to represent long-term memory, presynaptic potentials and neuron synchronized activation mechanism to realize identifying and recalling, and a time-delayed Hebb learning mechanism to fulfil the one-shot learning.

STAMN is an incremental, possibly looping, nonfully connected, asymmetric associative memory network. Nonhomogeneous nodes correspond to hidden state nodes and action nodes.

For the state node *j*, the input values are defined as follows: (1) the current observation activation value *b*_*j*_^*s*^(*t*), responsible for matching degree of state node *j*, and current observed value, which is obtained from the preprocessing neural networks (if the matching degree is greater than a threshold value, then *b*_*j*_^*s*^(*t*) = 1); (2) the observation activation value *b*_*j*_^*s*^(*t*) of the presynaptic nodes set *τ*_*j*_ of current state node *j*; (3) the identifying activation value *n*_*i*_^*s*^(*t*) of the previous state node *i* of state node *j*; (4) the activation value *n*_*i*_^*a*^(*t*) of the previous action node *i* of state node *j*. The output values are defined as follows: (1) The identifying activation value *n*_*j*_^*s*^(*t*) represents whether the current state *s* is identified to the hidden state *s*_*j*_ or not. (2) The transition prediction value *p*_*j*_^*s*^(*t*) represents whether the state node *j* is the current state transition prediction node or not.

For the action node *j*, the input values are defined as the current action activation value *b*_*j*_^*a*^(*t*), responsible for matching degree of action node *j* and current motor vectors. The output value is activation value of the action nodes *n*_*j*_^*a*^(*t*) and indicates that the action node *j* has been selected by agent to control the robot's current action.

For the STAMN, all nodes and connections weight do not necessarily exist initially. The weights *ω*_*ij*_(*t*) and ${\bar{\omega }}_{ij}(t)$ connected to state nodes can be learned by a time-delayed Hebb learning rules incrementally representing the LTM. The weight *w*_*ij*_(*t*) connected to action nodes can be learned by reinforcement learning. All nodes have activity self-decay mechanism to record the duration time of this node representing the STM. The output of the STAMN is the winner state node or winner action node by winner takes all.

**Figure 2:**
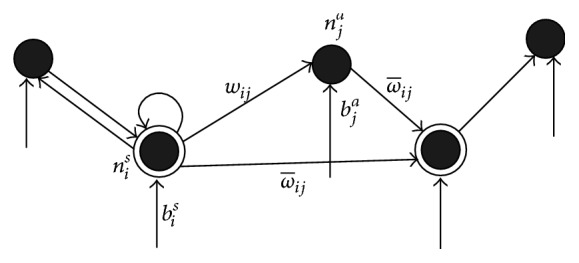
STAMN structure diagram.

STAMN architecture is shown in Figure 2, where black nodes represent action nodes and concentric circles nodes represent state nodes.

### 3.2. Short-Term Memory

We using self-decay of neuron activity to accomplish short-term memory, no matter whether observation activation *b*_*i*_^*s*^(*t*) or identifying activation *n*_*i*_^*s*^(*t*). The activity of each state node has self-decay mechanism to record the temporal order and the duration time of this node. Supposing the factor of self-decay is *γ*_*i*_^*s*^ ∈ (0,1) and the activation value *b*_*i*_^*s*^(*t*), *n*_*i*_^*s*^(*t*)∈[0,1] the self-decay process is shown as

(2) bist,nist=0bist,nist⩽ηγisbist−1,γisnist−1else.

The activity of action node also has self-decay characteristic. Supposing the factor of self-decay is *δ*_*i*_^*a*^ ∈ (0,1) and the activation value *b*_*i*_^*a*^(*t*), *n*_*i*_^*a*^(*t*)∈[0,1] the self-decay process is shown as

(3) biat,niat=0biat,niat⩽μδiabiat−1,δianiat−1else,

wherein *η* and *μ* are active thresholds and *γ*_*i*_^*s*^ and *δ*_*i*_^*a*^ are self-decay factors. Both determine the depth of short-term memory *k*, where *t* is a discrete time point by sampling at regular intervals Δ*t*, and Δ*t* is a very small regular interval.

### 3.3. Long-Term Memory

Long-term memory can be classified into semantic memory and episodic memory. Learning of history sequence is considered as the episodic memory, generally adopting the one-shot learning to realize. We use the time-delayed Hebb learning rules to fulfil the one-shot learning.

(*1) The k-Step Long-Term Memory Weight ω*_*ij*_(*t*). The weight *ω*_*ij*_(*t*) connected to state nodes *j* represents *k*-step long-term memory. This is a past-oriented behaviour in the STAMN. The weight *ω*_*ij*_(*t*) is adjusted according to

(4) $$\begin{array}{c} \underset{n_{j}^{s}\left(t\right)=1}{\underset{i\in \tau_{j}}{\omega_{ij}\left(t\right)}}=\left\{\begin{array}{cc} n_{j}^{s}\left(t\right)n_{i}^{X}\left(t\right) & \omega_{ij}\left(t-1\right)=0,\;X\in \left\{s,a\right\}\\ \omega_{ij}\left(t-1\right)+\alpha \left(n_{i}^{X}\left(t\right)-\omega_{ij}\left(t-1\right)\right) & \text{else}, \end{array}\right. \end{array}$$

where *j* is the current identifying activation state node, *n*_*j*_^*s*^(*t*) = 1. Because the identifying activation process is a neuron synchronized activation process, when *n*_*j*_^*s*^(*t*) = 1, all nodes whose *n*_*i*_^*s*^(*t*) and *n*_*i*_^*a*^(*t*) are not zero are the contextual information related to state node *j*. These nodes whose *n*_*i*_^*s*^(*t*) and *n*_*i*_^*a*^(*t*) are not zero are considered as the presynaptic node set of current state node *j*. The presynaptic node set of current state node *j* is expressed by *τ*_*j*_, where *n*_*i*_^*s*^(*t*), *n*_*i*_^*a*^(*t*) represent the activation value at time *t*, and *n*_*i*_^*s*^(*t*), *n*_*i*_^*a*^(*t*) record not only the temporal order but also the duration time because of the self-decay of neuron activity. If *n*_*i*_^*s*^(*t*), *n*_*i*_^*a*^(*t*) are smaller, the node *i* is more early to current state node *j*. *ω*_*ij*_(*t*) is activation weight between presynaptic node *i* and state node *j*. *ω*_*ij*_(*t*) records context information related to state node *j* to be used in identifying and recalling, where *ω*_*ij*_(*t*)∈[0,1], *ω*_*ij*_(0) = 0, and *α* is learning rate.

**Figure 3:**
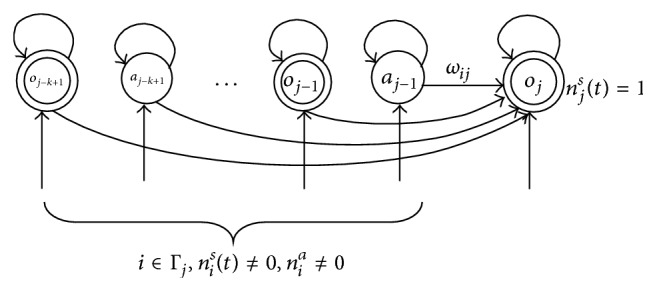
Past-oriented weights *ω*_*ij*_(*t*) update process associated with state node *j*.

The weight *ω*_*ij*_(*t*) is time-related contextual information; the update process is shown as in Figure 3.

(*2) One-Step Transition Prediction Weight* ${\bar{\omega }}_{ij}\left(t\right)$. The weight ${\bar{\omega }}_{ij}(t)$ connected to state nodes *j* represents one-step transition prediction in LTM. This is a future-oriented behaviour in the STAMN. Using the time-delayed Hebb learning rules, the weight ${\bar{\omega }}_{ij}(t)$ is adjusted according to

(5) $$\begin{array}{c} \underset{n_{j}^{s}\left(t\right)=1}{\underset{i=\arg\max n_{i}^{X}\left(t\right)}{{\bar{\omega }}_{ij}\left(t\right)}}=\left\{\begin{array}{cc} n_{j}^{s}\left(t\right)n_{i}^{X}\left(t\right) & {\bar{\omega }}_{ij}\left(t-1\right)=0,\;X\in \left\{s,a\right\}\\ {\bar{\omega }}_{ij}\left(t-1\right)+\beta \left(n_{i}^{X}\left(t\right)-{\bar{\omega }}_{ij}\left(t-1\right)\right) & \text{else.} \end{array}\right. \end{array}$$

The transition activation of current state node *j* is only associated with the previous winning state node and action node, where *j* is the current identifying activation state node, *n*_*j*_^*s*^(*t*) = 1. The previous winning state node and action node are presented by *i* = arg⁡max⁡*n*_*i*_^*X*^(*t*), *X* ∈ {*s*, *a*}, where $\bar{\omega }(t)\in [0,1]$ and $\bar{\omega }(0)=0$, *β* is learning rate.

**Figure 4:**
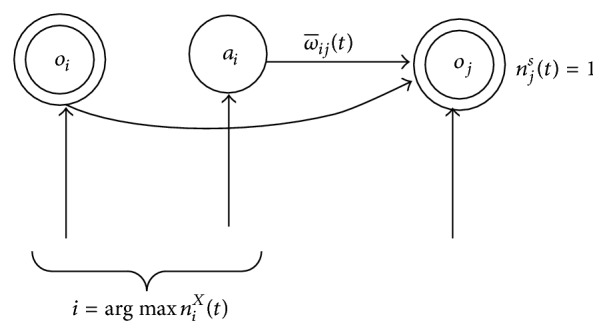
Future-oriented weights ${\bar{\omega }}_{ij}(t)$ update process associated with state node *j*.

The weight ${\bar{\omega }}_{ij}(t)$ is one-step transition prediction information; the update process is shown as in Figure 4. 

(*3) The Weight w*_*ij*_(*t*)* Connected to Action Nodes.* The activation of action node is only associated with the corresponding state node which selects this action node directly. We assume the state node with maximal identifying activation value is the state node *i* at the time *t*, so the connection weight *w*_*ij*_(*t*) connected to current selected action node *j* is adjusted by

(6) $$\begin{array}{c} \underset{n_{j}^{a}\left(t\right)=1}{\underset{i=\arg\max n_{i}^{s}\left(t\right)}{w_{ij}\left(t\right)}}=\left\{\begin{array}{cc} Q_{0}\left(s_{i},a_{j}\right) & w_{ij}\left(t-1\right)=0\\ -Q\text{\_max\_const} & a_{j}\text{ is a invalid action}\\ Q_{t}\left(s_{i},a_{j}\right) & \text{else}, \end{array}\right. \end{array}$$

where *w*_*ij*_(*t*) is represented by *Q* function *Q*_*t*_(*s*_*t*_, *a*_*j*_). This paper only discusses how to build generalized environment model, not learning the optimal policy, so this value is set to be the exhaustive exploration *Q* function based on the curiosity reward. The curiosity reward is described by (7). When action *a*_*j*_ is an invalid action, *w*_*ij*_(*t*) is defined to be a large negative constant, which is avoided going to the dead ends

(7) $$\begin{array}{c} r\left(\;t\;\right)=\left\{\begin{array}{cc} C-\frac{n_{t}}{n_{ave}} & n_{ave}>0\\ C & n_{ave}=0, \end{array}\right. \end{array}$$

where *C* is a constant value, *n*_*t*_ represents the count of exploration to the current action by the agent, and *n*_ave_ is the average count of exploration to all actions by the agent; *n*_*t*_/*n*_ave_ represents the degree of familiarity with the current selected action. The curiosity reward is updated when each action is finished. *Q*_*t*_(*s*_*i*_, *a*_*j*_) update equation is showed by (8), where *λ* is learning rate

(8) Qtsi,aj=1−λQt−1si,aj+λrti=arg⁡max⁡nist,  njat=1Qt−1si,ajelse.

**Figure 5:**
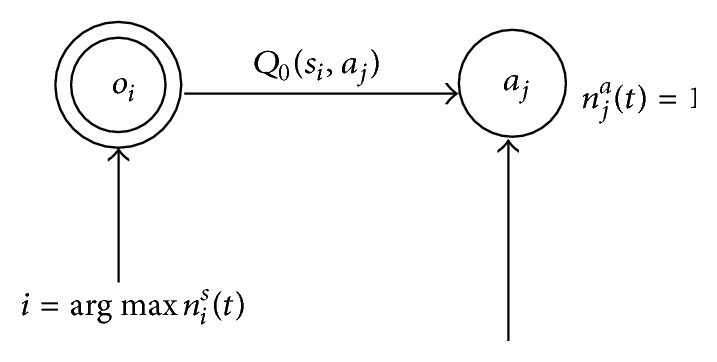
Weights *w*_*ij*_(*t*) updating process associated with action node *j*.

The update process is shown as in Figure 5.

The action node *j* is selected by *Q*_*t*_(*s*_*t*_, *a*_*j*_) according to

(9) $$\begin{array}{c} a_{j}=\underset{\begin{array}{c} j\in A_{i}^{\mathrm{vl}}\\ n_{i}^{s}\left(t\right)=1 \end{array}}{\arg\max\;}Q\left(\;s_{i},a_{j}\;\right), \end{array}$$

where *A*_*i*_^vl^ is the valid action set of state node *i*. *n*_*i*_^*s*^(*t*) = 1 represent that the sate node *i* is identified, and the action node *j* was selected by agent to control the robot's current action; set *n*_*i*_^*a*^(*t*) = 1.

### 3.4. The Constructing Process in STAMN

In order to construct the minimal looping hidden state transition model, we need to identify the looped state by identifying hidden state. There exist identifying phase and recalling phase (transition prediction phase) simultaneously in the constructing process in STAMN. There is a chicken and egg problem during the constructing process: the building of the STAMN depends on state identifying; conversely, state identifying depends on the current structure of the STAMN. Thus, exhaustive exploration and *k*-step variable memory length (depends on the prediction criterion) are used to try to avoid local minima that this interdependent causes.

According to Algorithm 12, The identifying activation value of state node *s*_*j*_ depends on three identifying processes: *k*-step history sequence identifying, following identifying, and previous identifying. We provide calculation equations for each identifying process.

(*1) k-Step History Sequence Identifying.* The matching degree of current *k*-step history sequence with the identifying history sequence *h*_*j*_ for *s*_*j*_ is computed to identify the looping state node *j*. First, we compute the presynaptic potential *V*_*j*_(*t*) for the state node *j* according to

(10) $$\begin{array}{c} V_{j}\left(\;t\;\right)=\underset{i\in \tau_{j}}{\sum }C_{ij}⌀\left(\;b_{i}^{X}\left(\;t\;\right),\omega_{ij}\;\right)\;X\in \left\{s,a\right\}, \end{array}$$

where *b*_*i*_^*X*^(*t*) is the current activation value in the presynaptic node set *τ*_*j*_. *C*_*ij*_ is the confidence parameter which means the node *i*'s importance degree in the presynaptic node set *τ*_*j*_. The value of *C*_*ij*_ can be set in advance, and ∑_*i*∈*τ*_*j*__ *C*_*ij*_ = 1. The function *⌀* represents the similar degree between activation value of the presynaptic node and the contextual information *ω*_*ij*_ in LTM. *⌀*(*x*, *y*)∈[0,1]; the similar degree is high between *x* and *y*; thus *⌀*(*x*, *y*) is close to 1. According to “winner takes all,” among all nodes whose presynaptic potentials exceed the threshold *ϑ*, the node with the largest presynaptic potential will be selected.

The presynaptic potential *V*_*j*_(*t*) represents the synchronous activation process of presynaptic node set of *τ*_*j*_, which represents the previous *k* − 1 step contextual information matching of state node *j*. To realize all *k*-step history sequence matching, the *k*-step history sequence identifying activation value of the state node *j* is given below:

(11) mjst=HVjt,ϑ·HVjt,maxk:1→Nc⁡VktbjstHx,y=0x<y1x≥y,

where max_*k*:1→*N*^*c*^_(*V*_*k*_(*t*)) is the maximum potential value of node *k*. *H*(*V*_*j*_(*t*), *ϑ*) · *H*(*V*_*j*_(*t*), max_*k*:1→*N*^*c*^_(*V*_*k*_(*t*))) means the node with the largest potential value is selected among all nodes whose synaptic potentials exceed the threshold *ϑ*. *b*_*j*_^*s*^(*t*) represents the matching degree of state node *j* and current observed value. If *m*_*j*_^*s*^(*t*) = 1, the current state *s* is identified to looped state node *j* by the *k* step memory.

(*2) Following Identifying.* If the current state *s* is identified as the state *s*_*i*_, then the next transition prediction state is identified as the state *s*_*j*_ = *f*_*s*_(*s*_*i*_, *a*_*i*_). First, we compute the transition prediction value for the state node *j* according to

(12) $$\begin{array}{c} p_{j}^{s}\left(\;t\;\right)=\underset{i=\arg\max n_{i}^{X}\left(t\right)}{\sum }D_{ij}\phi \left(\;n_{i}^{X}\left(\;t\;\right),{\bar{\omega }}_{ij}\;\right)\;X\in \left\{s,a\right\}, \end{array}$$

where *n*_*i*_^*X*^(*t*) is the identifying activation value of the state node and action node at time *t*. *i* = arg⁡max⁡*n*_*i*_^*X*^(*t*) indicates state node *i* and action node *i* are the previous winner nodes. *D*_*ij*_ is the confidence parameter which means the node *i*'s importance degree. The value of *D*_*ij*_ can be set in advance, and ∑_*i*=arg⁡max⁡*n*_*i*_^*X*^(*t*)_ *D*_*ij*_ = 1. ${\bar{\omega }}_{ij}$ records one-step transition prediction information related to state node *j* to be used in identifying and recalling phase. If the current state *s* is identified as the hidden state *s*_*i*_, *p*_*j*_^*s*^(*t*) represent the probability of the next transition prediction state is *j*.

If the next prediction state node *j* is the same as the current observation value, the current history sequence is identified as the state node *j*. the following identifying value of the state node *j* is given below:

(13) qjst=Hpjst,ϑ·Hpjst,maxk:1→Nc⁡pkstbjst.

If *q*_*j*_^*s*^(*t*) = 1, the current history sequence *h*_*t*_ is identified to looped state node *j* by the following identifying. If *p*_*j*_^*s*^(*t*) ≥ max_*k*:1→*N*^*c*^_(*p*_*k*_^*s*^(*t*)), *p*_*j*_^*s*^(*t*) ≥ *ϑ*, and *q*_*j*_^*s*^(*t*) ≠ 1, then there exists *b*_*j*_^*s*^(*t*) ≠ 1, representing mismatching of state node *j* and current observed value. There exist trans(*h*_*t*_, *a*_*i*_)≠ trans(*h*_*i*_, *a*_*i*_). According to transition prediction criterion (Algorithm 13), the current history sequence *h*_*t*_ is not identified by the hidden sate *s*_*i*_, so the identifying history sequence length for *h*_*t*_ and *h*_*i*_ separately must increase to *k* + 1.

(*3) Previous Identifying.* If the current history sequence is identified as the state *s*_*j*_, then the previous state *s*_*i*_ is identified such that *s*_*j*_ = *f*_*s*_(*s*_*i*_, *a*_*i*_). First, we compute the identifying activation value for all states *s*. if there exists *n*_*j*_^*s*^(*t*) = 1, then the current state *s*_*t*_ is identified as state *s*_*j*_, and the previous state *s*_*t*−1_ is identified as the previous state *s*_*i*_ of state *s*_*j*_. The previous identifying value of the state node *j* is defined as *d*_*i*_^*s*^(*t*). The previous state *s*_*i*_ is *i* = arg max(*b*_*i*_^*s*^(*t*)) satisfying condition 1 and condition 2. Then we set *d*_*i*_^*s*^(*t*):

 Condition 1:
  (14) $$\begin{array}{c} n_{j}^{s}\left(\;t\;\right)=1 \end{array}$$
 Condition 2:
  (15) $$\begin{array}{c} \phi \left(\;b_{i}^{s}\left(\;t\;\right),{\bar{\omega }}_{ij}\;\right)>\vartheta \end{array}$$

According to above three identifying processes, the identifying activation value of the state node *j* is defined as follows:

(16) $$\begin{array}{c} n_{j}^{s}\left(\;t\;\right)=\max\left(\;m_{j}^{s}\left(\;t\;\right),q_{j}^{s}\left(\;t\;\right),d_{j}^{s}\left(\;t\;\right)\;\right). \end{array}$$

According to Algorithm 11, we give Pseudocode 1 for Algorithm 11.

The pseudocode for Algorithm 12 is as follows: the current history sequence identifying algorithm (identifying phase). Compute the identifying activation value according to (16).

The pseudocode for Algorithm 13 is in Pseudocode 2.

## 4. Simulation

### 4.1. A Simple Example of Deterministic POMDP

**Figure 6:**
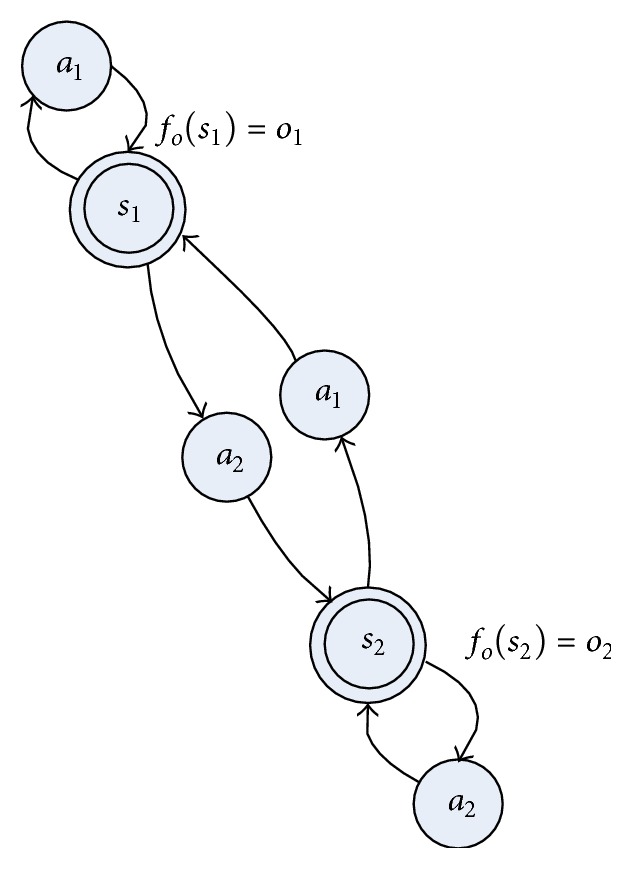
The STAMN model with memory depth 1.

We want to construct the looping transition model as in Figure 1. First, we obtain the transition instance *h*_*t*_ : {*o*_1_, *a*_1_, *o*_1_, *a*_2_, *o*_2_, *a*_2_, *o*_2_, *a*_1_, *o*_1_, *a*_1_, *o*_1_}. According to Algorithm 11, set the initial identifying history sequence length *k* = 1. The STAMN model is constructed incrementally using *h*_*t*_ as in Figure 6.

When *h*_*t*_ = {*o*_1_, *a*_1_, *o*_1_, *a*_2_, *o*_2_, *a*_2_, *o*_2_, *a*_1_, *o*_1_, *a*_1_, *o*_1_, *a*_2_, *o*_1_}, because of *k* = 1, the looped state is identified by the current observation vector. Thus *h*_1_ = {*o*_1_} and *h*_2_ = {*o*_1_} are the identifying history sequences for the state *s*_1_. Since *o*_1_ = tans(*h*_1_, *a*_2_) and *o*_2_ = tans(*h*_2_, *a*_2_) in *h*_*t*_ exist, so as to trans(*h*_1_, *a*_2_)≠ trans(*h*_2_, *a*_2_), according to Lemma 9, *h*_1_ and *h*_2_ are the identifying history sequences for the states *s*_*i*_ and *s*_*j*_, respectively, and *s*_*i*_ ≠ *s*_*j*_. So *o*_1_ is distinguished as *o*_1_′ and *o*_1_′′. And *h*_*t*_ = {*o*_1_, *a*_1_, *o*_1_′, *a*_2_, *o*_2_, *a*_2_, *o*_2_, *a*_1_, *o*_1_, *a*_1_, *o*_1_′′, *a*_2_, *o*_1_}.

According the Lemma 10, for any two identifying history sequences *h*_*i*_ and *h*_*j*_ separately for *o*_1_′ and *o*_1_′′, there exists *h*_*i*_ ≠ *h*_*j*_ iff *s*_*i*_ ≠ *s*_*j*_. So the identifying history sequence length for *h*_*i*_ and *h*_*j*_ separately for *s*_*i*_ and *s*_*j*_ must increase to 2. For *o*_1_′, the identifying history sequence is {*o*_1_, *a*_1_, *o*_1_′}, and for *o*_1_′′, the identifying history sequence is {*o*_1_, *a*_1_, *o*_1_′′}. These two identifying history sequences are identical, so longer transition instance is needed.

When *h*_*t*_ = {*o*_1_, *a*_1_, *o*_1_′, *a*_2_, *o*_2_, *a*_2_, *o*_2_, *a*_1_, *o*_1_, *a*_1_, *o*_1_′′, *a*_2_, *o*_1_′, *a*_2_, *o*_2_, *a*_1_, *o*_1_′, *a*_2_, *o*_2_, *a*_1_, *o*_1_, *a*_1_, *o*_1_, *a*_1_, *o*_1_′, *a*_2_, *o*_2_}. In *h*_*t*_, we obtain the identifying history sequence *h*_*i*_ ≠ *h*_*j*_ for states *o*_1_′ and *o*_1_′′. For *k* = 2, the distinguished identifying history sequence for *o*_1_′ is {*o*_1_′′, *a*_2_, *o*_1_′} and {*o*_2_, *a*_1_, *o*_1_′}. However, the distinguished identifying history sequence for *o*_1_′′ is {*o*_1_, *a*_1_, *o*_1_′′}, which is not a discriminated history sequence from the identifying history sequence for *o*_1_′. So the identifying history sequence length for *o*_1_′′ must increase to 3. The identifying history sequences for *o*_1_′ and *o*_1_ are described as in Table 1.

According to *k*-step history identifying, following identifying and previous identifying, *h*_*t*_ can be represented as *h*_*t*_ = {*o*_1_′′, *a*_1_, *o*_1_′, *a*_2_, *o*_2_, *a*_2_, *o*_2_, *a*_1_, *o*_1_′, *a*_1_, *o*_1_′′, *a*_2_, *o*_1_′, *a*_2_, *o*_2_, *a*_1_, *o*_1_′, *a*_2_, *o*_2_, *a*_1_, *o*_1_′, *a*_1_, *o*_1_′′, *a*_1_, *o*_1_′, *a*_2_, *o*_2_}.

**Figure 7:**
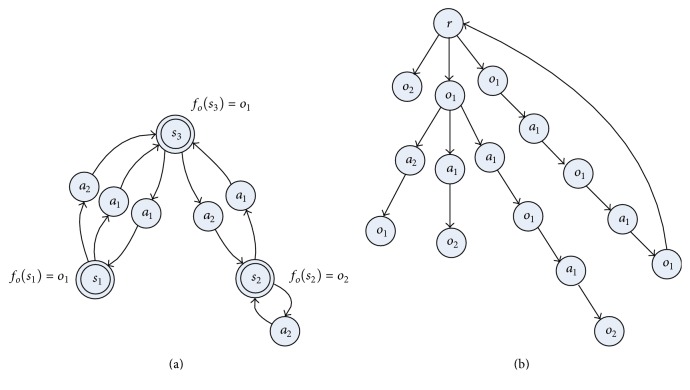
(a) The STAMN model. (b) The LPST model.

According to Pseudocode 1, the STAMN model is constructed incrementally using *h*_*t*_ as in Figure 7(a), and the LPST is constructed as Figure 7(b). The STAMN has the fewest nodes because the state nodes in STAMN represent the hidden state, and the state nodes in LPST represent the observation state.

**Figure 8:**
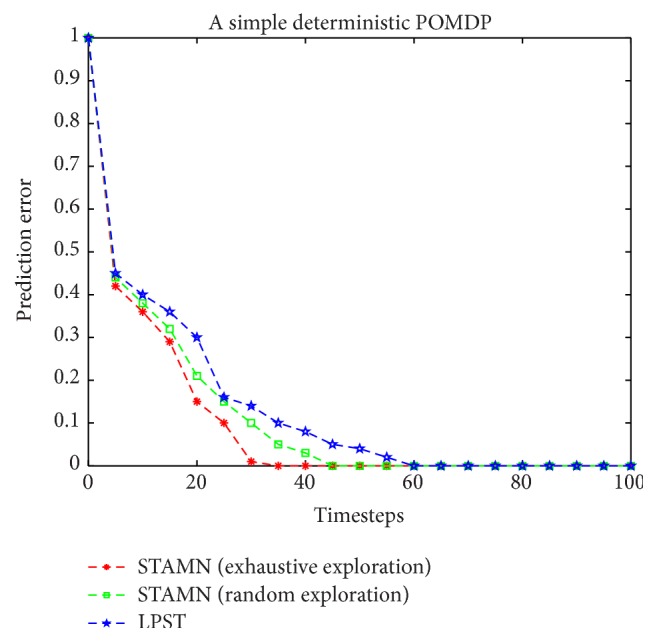
Comparative performance results for a simple deterministic POMDP.

To illustrate the difference between the STAMN and LPST, we present the comparison results in Figure 8.

After 10 timesteps, the algorithms use the current learned model to realize the transition prediction, and the transition prediction criterion is expressed by the prediction error. The data point represents the average prediction error over 10 runs. We ran three algorithms: the STAMN with exhaustive exploration, the STAMN with random exploration, and the LPST.

Figure 8 shows that three algorithms all can produce the correct model with zero prediction error at last because of no noise. However, the STAMN with exhaustive exploration has the better performance and the faster convergence speed because of the exhaustive exploration *Q*-function.

### 4.2. Experimental Comparison in 4*∗*3 Grid Problem

**Figure 9:**
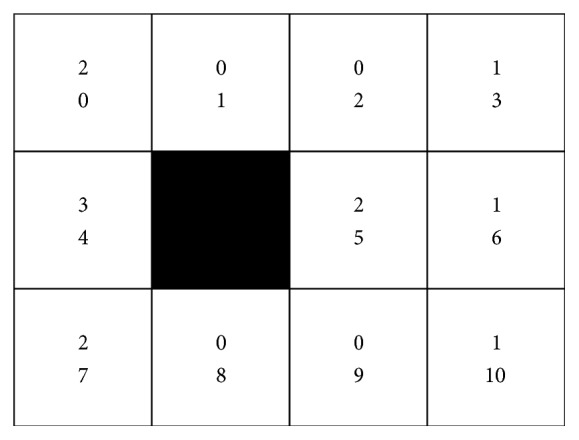
4*∗*3 gird problem.

First, we use the small POMDP problem to test the feasibility of the STAMN. 4*∗*3 gird problem is selected, which is shown in Figure 9. The agent wanders inside the grid and has only left sensor and right sensor to report the existence of a wall in the current position. The agent has four actions: forward, backward, turn left, and turn right. The reference direction of the agent is northward.

**Figure 10:**
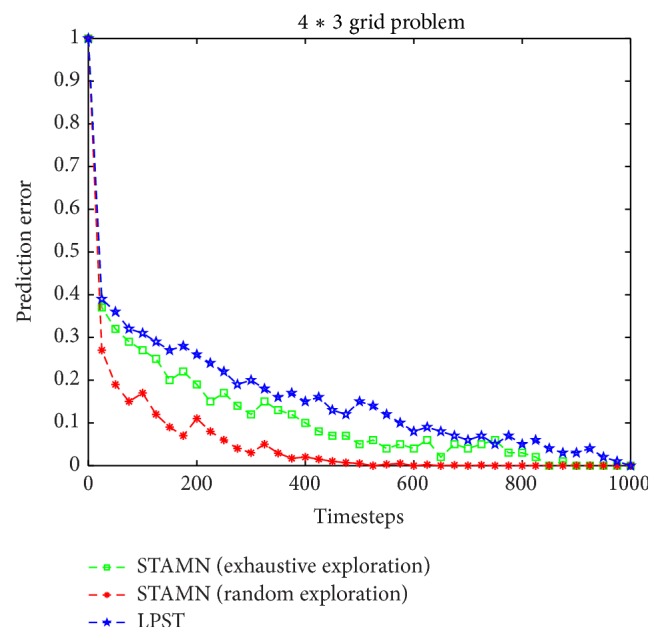
Comparative performance results for 4*∗*3 gird problem.

In the 4*∗*3 gird problem, the hidden state space size |*S*| is 11, the action space size |*A*| is 4 for each state, and the observation space size |*O*| is 4. The upper figure in the grid represents the observation state and the bottom figure in the grid represents the hidden state. The black grid and the wall are regarded as the obstacles. We present a comparison between the LPST and the STAMN. The results are shown in Figure 10. Figure 10 shows that the STAMN with exhaustive exploration still has the better performance and the faster convergence speed in the 4*∗*3 gird problem.

The number of state nodes and action nodes in STAMN and LPST is described in Table 2. In STAMN, the state node represents the hidden state, but in LPST, the sate node represents observation value, and the hidden state is expressed by *k*-step past observations and actions; thus, more observation nodes and action nodes are repeated created, and most of the observation nodes and action nodes are the same. LPST has the perfect observation prediction capability and is the same as the STAMN but is not the state transition model.

In STAMN, The setting of parameters *μ*, *η*, *γ*_*i*_^*s*^, *δ*_*i*_^*a*^ is very important which determines the depth *k* of short-term memory. We assume *γ*_*i*_^*s*^ = *δ*_*i*_^*a*^ = 0.9 and *μ* = *η* = 0.9 initial representing *k* = 1. When we need to increase *k* to *k* + 1, we only need to decrease *μ*, *η* according to *η* = *η* − 0.2, *μ* = *μ* − 0.1. The other parameters are determined relative easily. We set learning rates *α* = *β* = *γ* = 0.9 and set the confidence parameters *C*_*ij*_ = 1/|*τ*_*j*_|, *D*_*ij*_ = 1/2 representing the same importance degree. Set constant values *C* = 5, *ϑ* = 0.9.

### 4.3. Experimental Results in Complex Symmetrical Environments

**Figure 11:**
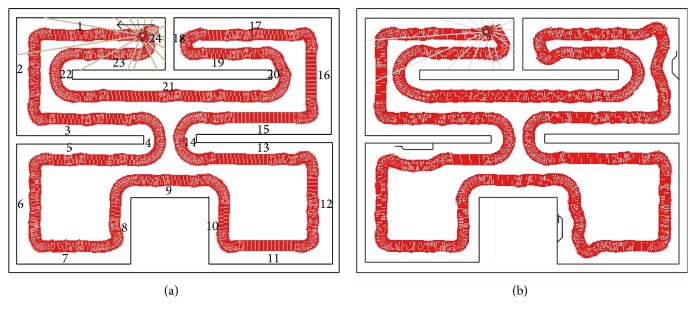
(a) Complex symmetrical simulation environment. (b) The disturbed environment.

The symmetrical environment in this paper is very complex, which is shown in Figure 11(a). The robot wanders in the environment. By following wall behaviour the agent can recognize the left wall, right wall, and corridor landmarks. These observation landmarks are different from the observation vector in the previous grid problem; every observation landmark has different duration time. For analysis of the fault tolerance and robustness of STAMN, we assume the disturbed environment is shown in Figure 11(b).

**Figure 12:**
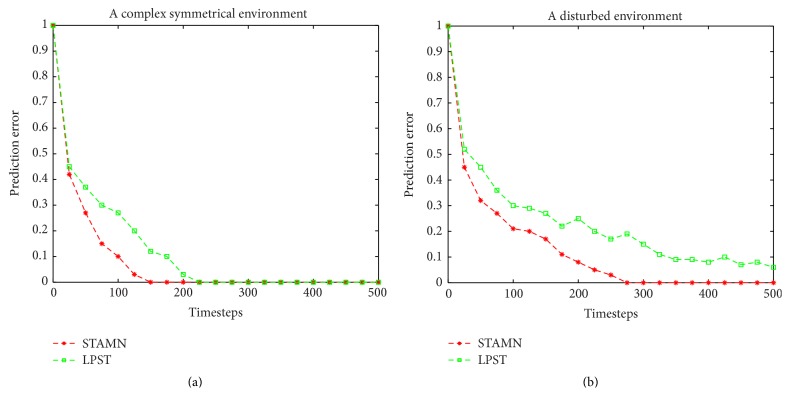
(a) Comparative results for a noise-free environment. (b) Comparative results for a disturbed environment.

The figures in Figure 11(a) represent the hidden states. The robot has four actions: forward, backward, turn left, and turn right. The initial orientation of the robot is shown in Figure 11(a). Since the robot follows the wall in the environment, the robot has only one optional action in each state. The reference direction of action is the robot current orientation. Thus, the path 2-3-4-5-6 and path 12-13-14-15-16 are identical, and the memory depth *k* = 6 is necessary to identify hidden states reliably. We present a comparison between the STAMN and the LPST in noise-free environment and disturbed environment. The results are shown as in Figures 12(a) and 12(b).  Figure 12(b) shows that STAMN has better noise tolerance and robustness than LPST because of the neuron synchronized activation mechanism, which bears the fault and noise in the sequence item duration time, and can realize the reliable recalling. However, the LPST cannot realize the correct convergence in reasonable time because of accurate matching.

## 5. Discussion

In this section, we compare the related work with the STAMN model. The related work mainly includes the LPST and the AMN.

### 5.1. Looping Prediction Suffix Tree (LPST)

LPST is constructed incrementally by expanding branches until they are identified or become looped using the observable termination criterion. Given a sufficient history, this model can correctly capture all predictions of identifying histories and can map the all infinite identifying histories onto a finite LPST.

The STAMN proposed in this paper is similar to the LPST. However, the STAMN is looping hidden state transition model, so in comparison with LPST, STAMN have less state nodes and action nodes because these nodes in LPST are based on observation, not hidden state. Furthermore, STAMN has better noise tolerance and robustness than LPST. The LPST realizes recalling by successive accurate matching, which is sensitive to noise and fault. The STAMN offers the neuron synchronized activation mechanism to realize recalling. Even if in noisy and disturbed environment, STAMN can still realize the reliable recalling. Finally, the algorithm for learning a LPST is an additional computation model, which is not a distributed computational model. The STAMN is a distributed network and uses the synchronized activation mechanism, where performance cannot become poor with history sequences increasing and scale increasing.

### 5.2. Associative Memory Networks (AMN)

STAMN is proposed based on the development of the associative memory networks. In existing AMN, firstly, almost all models are unable to handle complex sequence with looped hidden state. The STAMN realizes the identifying the looped hidden state indeed, which can be applied to HMM and POMDP problems. Furthermore, most AMN models can obtain memory depth determined by experiments. The STAMN offers a self-organizing incremental memory depth learning method, and the memory depth *k* is variable in different parts of state space. Finally, the existing AMN models general only record the temporal orders with discrete intervals Δ*t* rather than sequence items duration with continuous-time. STAMN explicitly deals with the duration time of each item.

## 6. Conclusion and Future Research

POMDP is the long-standing difficult problem in the machine learning. In this paper, SATMN is proposed to identify the looped hidden state only by the transition instances in deterministic POMDP environment. The learned STAMN is seen as a variable depth *k*-Morkov model. We proposed the heuristic constructing algorithm for the STAMN, which is proved to be sound and complete given sufficient history sequences. The STAMN is real self-organizing incremental unsupervised learning model. These transition instances can be obtained by the interaction with the environment by the agent and can be obtained by a number of training data that does not depend on the real agent. The STAMN is very fast, robust, and accurate. We have also shown that STAMN outperforms some existing hidden state methods in deterministic POMDP environment. The STAMN can generally be applied to almost all temporal sequences problem, such as simultaneous localization and mapping problem (SLAM), robot trajectory planning, sequential decision making, and music generation.

We believe that the STAMN can serve as a starting point for integrating the associative memory networks with the POMDP problem. Further research will be carried out on the following aspects: how to scale our approach to the stochastic case by heuristic statistical test; how to incorporate with the reinforcement learning to produce a new distributed reinforcement learning model; and how it should be applied to robot SLAM to resolve the practical navigation problem.

## Figures and Tables

**Pseudocode 1:**
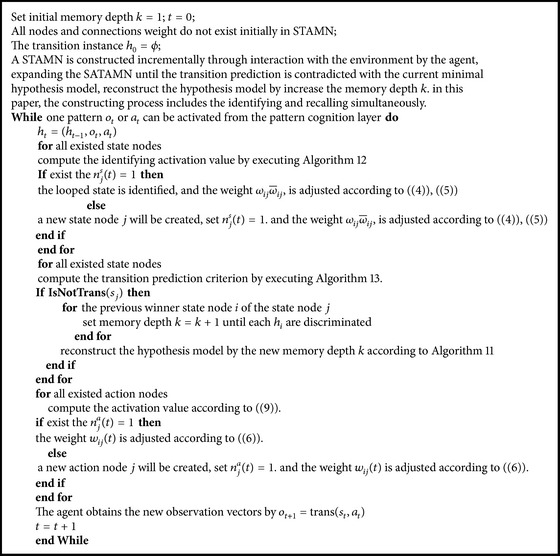
Algorithm for heuristic constructing the STAMN.

**Pseudocode 2:**
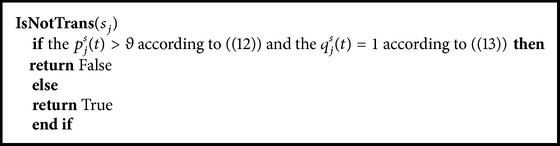
Transition prediction criterion algorithm (recalling phase).

**Table 1:** The identifying history sequences for *o*_1_′ and *o*_1_′′.

Hidden state	*o*_1_′ (*k* = 2)	*o*_1_′′ (*k* = 3)

Identifying history sequence	*o*_1_, *a*_1_, *o*_1_′	*o*_2_, *a*_1_, *o*_1_, *a*_1_, *o*_1_′′
	*o*_1_′′, *a*_2_, *o*_1_′	
	*o*_2_, *a*_1_, *o*_1_′	

**Table 2:** The number of state nodes and action nodes in STAMN and LPST.

Algorithm	The number of state nodes	The number of action nodes
LPST	70	50
STAMN	11	44
